# Retrospective evaluation of the predictive value of tumour burden at baseline [^68^ Ga]Ga-DOTA-TOC or -TATE PET/CT and tumour dosimetry in GEP-NET patients treated with PRRT

**DOI:** 10.1186/s41824-024-00210-y

**Published:** 2024-08-08

**Authors:** Anni Gålne, Anna Sundlöv, Olof Enqvist, Katarina Sjögreen Gleisner, Erik Larsson, Elin Trägårdh

**Affiliations:** 1 Department of Medical Imaging and Physiology, Skåne University Hospital, Lund and Malmö, Sweden; 2 Department of Translational Medicine, Lund University, Malmö, Sweden; 3 WCMM Wallenberg Centre for Molecular Medicine, Lund, Sweden; 4 Department of Clinical Sciences, Oncology and Pathology, Skåne University Hospital, Lund University, Lund, Sweden; 5 Eigenvision AB, Malmö, Sweden; 6 Department of Electrical Engineering, Chalmers University of Technology, Gothenburg, Sweden; 7 Medical Radiation Physics, Lund University, Lund, Sweden; 8 Department of Radiation Physics, Skåne University Hospital, Lund, Sweden

## Abstract

**Purpose:**

There is a lack of validated imaging biomarkers for prediction of response to peptide receptor radionuclide therapy (PRRT). The primary objective was to evaluate if tumour burden at baseline PET/CT could predict treatment outcomes to PRRT with [^177^Lu]Lu-DOTA-TATE. Secondary objectives were to evaluate if there was a correlation between tumour burden and mean tumour absorbed dose (AD) during first cycle, and if mean tumour AD or the relative change of tumour burden at first follow-up PET/CT could predict progression free survival (PFS) or overall survival (OS).

**Methods:**

Patients with gastroenteropancreatic neuroendocrine tumour (GEP-NET) treated with [^177^Lu]Lu-DOTA-TATE PRRT were retrospectively included. Tumour burden was quantified from [^68^ Ga]Ga-DOTA-TOC/TATE PET/CT-images at baseline and first follow-up and expressed as; whole-body somatostatin receptor expressing tumour volume (SRETVwb), total lesion somatostatin receptor expression (TLSREwb), largest tumour lesion diameter and highest SUVmax. The relative change of tumour burden was evaluated in three categories. Mean tumour AD was estimated from the first cycle of PRRT. PFS was defined as time from start of PRRT to radiological or clinical progression. OS was evaluated as time to death. Kaplan Meier survival curves and log-rank test were used to compare PFS and OS between different groups.

**Results:**

Thirty-one patients had a baseline PET/CT < 6 months before treatment and 25 had a follow-up examination. Median tumour burden was 132 ml (IQR 61–302) at baseline and 71 ml (IQR 36–278) at follow-up. Twenty-two patients had disease progression (median time to progression 17.2 months) and 9 patients had no disease progression (median follow-up 28.7 months). SRETVwb dichotomized by the median at baseline was not associated with longer PFS (*p* = 0.861) or OS (*p* = 0.937). Neither TLSREwb, largest tumour lesion or SUVmax showed significant predictive value. There was a moderately strong correlation, however, between SUVmax and mean tumour AD r = 0.705, *p* < 0.001, but no significant correlation between SRETVwb nor TLSREwb and mean tumour AD. An increase of SRETVwb, TLSREwb or largest tumour lesion at first follow-up PET/CT was significantly correlated with shorter PFS/OS.

**Conclusion:**

Tumour burden at baseline showed no predictive value of PFS/OS after PRRT in this small retrospective study. An increase of tumour burden was predictive of worse outcome.

**Supplementary Information:**

The online version contains supplementary material available at 10.1186/s41824-024-00210-y.

## Background

Gastroenteropancreatic neuroendocrine tumours (GEP-NET) are often indolent but frequently metastasized at diagnosis and curative surgery therefore often not possible (Plöckinger et al. 2004). Most GEP-NETs overexpresses the somatostatin receptor (SSTR) (Theodoropoulou and Stalla 2013). The SSTR is a target for both diagnostics with radiolabelled somatostatin analogues for SSTR positron emission tomography/computed tomography (PET/CT) (Johnbeck et al. 2014) and treatment with peptide receptor radionuclide therapy (PRRT) (Reubi 2003). Commonly used diagnostic positron-emitting radiolabelled somatostatin analogues are [^68^ Ga]Ga-DOTA-TOC and [^68^ Ga]Ga-DOTA-TATE (SSTR PET/CT) (Bozkurt et al. 2017). A high tumour uptake of these SSTR-binding tracers is required for treatment with [^177^Lu]Lu-DOTA-TATE PRRT. A recent review and meta analyses concluded that the tumour burden measured at SSTR PET/CT had high correlation with PFS and OS and is of importance for prognostication (Hou et al. 2021). However, it is important to note that these studies were conducted under diverse conditions, with patients receiving varying types of treatments or surgeries. Additionally, significantly different cut-offs for tumour volume were suggested (Hou et al. 2021). Another recent review concluded that there are several known prognostic factors such as tumour burden, grade and [^18^F]Fluorodeoxyglucose (FDG) uptake at PET/CT, but there are still no validated imaging biomarkers that are able to predict treatment outcomes of PRRT (Albertelli et al. 2021). In conclusion, there is a need for validated SSTR PET/CT imaging biomarkers for selecting patients for PRRT by predicting the treatment effect.

One potential explanation to why tumour burden may predict treatment outcomes after PRRT is that differences in tumour burden may result in differences in mean absorbed dose (AD) to the tumours. The tumour AD varies between tumour lesions and changes during repeated treatment cycles (Roth et al. 2021). Theoretically, a larger tumour burden could impact the amount of available SSTR for [^177^Lu]Lu-DOTA-TATE, leading to a lower activity concentration in the tumours and, consequently, a lower mean AD to the lesions. If a large tumour burden results in a lower mean tumour AD this could lead to a shorter PFS/OS during standard treatment with [^177^Lu]Lu-DOTA-TATE. A change in tumour burden or receptor expression might also impact the mean tumour AD from cycle to cycle.

The primary objective of this study was to evaluate if tumour burden defined as whole-body somatostatin receptor expressing tumour volume (SRETVwb) and whole-body total lesion somatostatin receptor expression (TLSREwb), largest tumour lesion diameter or highest SUVmax could predict PFS or OS after treatment with [^177^Lu]Lu-DOTA-TATE. Secondary objectives were to evaluate if there is a correlation between SRETVwb, TLSREwb or SUVmax and mean tumour AD, or if mean tumour AD could predict PFS or OS. In a sub-group of patients who performed SSTR PET/CT both before PRRT and at follow-up, other secondary objectives were to evaluate if the change in tumour burden could predict PFS or OS after treatment with [^177^Lu]Lu-DOTA-TATE. An exploratory objective was to evaluate if the tumour burden in patients receiving PRRT could be accurately evaluated with an in-house developed AI model (Gålne et al. 2023).

## Patients and methods

### Patient enrolment

**Fig. 1:**
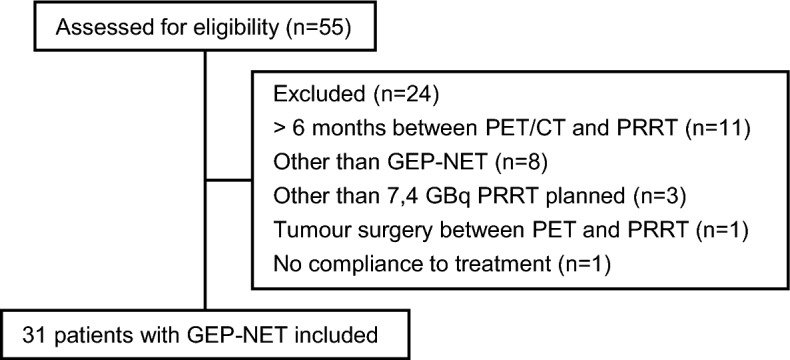
Consort diagram of the retrospective study

All adults ≥ 18 years old, starting treatment with [^177^Lu]Lu-DOTA-TATE at Skåne University Hospital between 2018-12-01 and 2021-10-22 were retrospectively assessed for inclusion in this analysis. Patients were excluded if they did not have a PET/CT done < 6 months before treatment. Patients were also excluded if they had other tumour than GEP-NET or other than standard treatment (7.4 GBq × 4) planned. Two more patients were excluded, one due to surgery between PET/CT and PRRT and one due to non-compliance to treatment. The CONSORT diagram (Consolidated Standards of Reporting Trials) provides further details (Fig. 1). The patients had histologically confirmed NET, unresectable disease and confirmed progression of disease before PRRT, except for two patients who had symptomatic, widespread disease at diagnosis. Follow-up visits and radiology was performed in the clinical setting, customized after the patients’ requirements. Data on age, sex, other diseases, histopathological diagnosis, Ki-67 index, type of earlier or ongoing treatment, blood results, WHO performance and clinical status at diagnosis and during follow-up were collected by reviewing the patient’s digital medical record. The study was approved by the Swedish Ethical Review Authority (Dnr 2019–00411 and 2021-04197). As the study was retrospective, informed consent was not needed according to the ethical approval.

### Treatment

[^177^Lu]Lu-DOTA-TATE was administered according to institutional guidelines with 4 cycles planned with a 8 week interval and a fixed activity of 7.4 GBq. Kidneys were protected with a reno-protective amino acid infusion which started 30 min before the infusion of [^177^Lu]Lu-DOTA-TATE and continued for a total of 6 h. Planned for day 4 after administration of the first treatment cycle, single photon emission computed tomography/computed tomography (SPECT/CT) was acquired using a GE Discovery 670 NM SPECT/CT (GE Healthcare, Milwaukee, USA). Two field of views were acquired, covering roughly 77 cm over the torso, and used for kidney dosimetry and evaluation of tumour uptake.

### PET/CT

The PET/CT scans were acquired using a Discovery MI or Discovery D690 (GE Healthcare, Milwaukee, USA) PET/CT system. Both [^68^ Ga]Ga-DOTA-TATE and [^68^ Ga]Ga-DOTA-TOC are included in this analysis due to a shift in production during 2019. The patient preparation, radiotracer production and PET/CT acquisition were the same as earlier described (Gålne et al. 2023). Together with the PET scan either a low-dose CT scan or a diagnostic CT with intravenous contrast (if there were no contraindications) was performed for attenuation correction and anatomic correlation. If the CT scan performed with the PET was regarded as too old as a baseline CT, a new contrast-enhanced baseline CT was performed before treatment.

### Quantification of tumour burden

In each patient, tumour burden was evaluated at baseline PET/CT and for a sub-group of 25 patients who had a PET/CT done as follow-up the change of tumour burden was re-evaluated. For 23 patient the follow-up PET/CT was obtained after end of treatment with PRRT and for two patients the PET/CT was done during treatment. For both patients this examination was acquired after the third cycle after which they received the last cycle of PRRT. Manual measurements of SRETVwb was performed with the semiautomatic delineation of the tumour volume of more than 50% of SUVmax, described in detail earlier (Gålne et al. 2023; Ohlsson et al. 2022). TLSREwb was defined as the sum of all products of the SUVmean and the volume (ml) of each lesion, as described earlier (Gålne et al. 2023).The highest SUVmax in any tumour lesion was extracted from the SRETVwb and the largest lesion diameter were measured from the most recent baseline examination and the first follow-up examination after treatment. Tumour burden was also automatically quantified with the AI model developed earlier (Gålne et al. 2023). SRETVwb and TLSREwb were automatically derived from baseline PET/CT and follow-up PET/CT by the AI model. Connected tumour components were classified as lesions by the AI model as earlier described (Gålne et al. 2023) and the largest lesion volume and the highest SUVmax in any tumour lesion could automatically be derived. One PET/CT examination was interrupted halfway and then re-started, with part of liver lesions imaged twice. These images were handled as two separate examinations by the AI model and lesions marked twice were removed (173 ml) and the rest of the lesions from the two sets of images were summarized as one examination in the analysis. For 3 patients the AI model segmented obvious false positive lesions (extravasation and segmentation of the bladder), these lesions were removed, median 1.7 ml (range 0.9–34.8 ml). Missed lesions were not added.

### Dosimetry

Quantitative [^177^Lu]Lu-DOTA-TATE SPECT/CT images were reconstructed following previously described procedures, including compensations for attenuation, scatter, and collimator response (Sundlöv et al. 2018). Image segmentation was performed using a semi-automatic method for fast tumour segmentation currently under development within the LundADose software (Ljungberg and Sjögreen 2018). Briefly, the method was based on the construction of a difference of Gaussians filtering pyramid, to which a region-growing algorithm was applied with automatically generated seeds (Sjögreen Gleisner et al. 2020). Segmented regions were visually inspected by the responsible radiologist and, when required, complemented by manual segmentation and removal of falsely segmented physiological uptakes. Activity concentrations for individual tumours were calculated from the activity and volume of each segmented region and application of a volume-dependent recovery correction (Roth et al. 2021). Tumour ADs were calculated by the assumption of an effective half-life of 103 h for grade 1 NETs, and 81 h for grade 2 NETs (Roth et al. 2021), and local energy deposition of the emitted electron energy from ^177^Lu (Sjögreen Gleisner et al. 2022). The mean AD across tumours in a patient was taken as the mass-weighted mean, i.e. by weighting individual tumour ADs by their respective mass (Gustafsson et al. 2023).

### Evaluation of PFS/OS

PFS was evaluated as time from first cycle of [^177^Lu]Lu-DOTA-TATE to radiological progression according to RECIST1.1 criteria at follow-up contrast enhanced CT scans compared with baseline CT or clinical/biochemical progression as determined by the treating physician. OS was evaluated as death by any cause. Patients were followed until the date for progression of disease, death or until 2023–03-30.

### Statistical analysis

IBM SPSS Statistics version 29 was used for all statistical analyses. Descriptive statistics were used for patient characteristics. Manual measurements of SRETVwb, TLSREwb, highest SUVmax in any tumour lesion and largest lesion diameter as well as mean tumour AD were dichotomized by the median and Kaplan Meier survival curves and log rank test were used to compare both PFS and OS between the groups. For the manual measurements of tumour burden and mean tumour AD, univariate Cox regression analyses for evaluation of the relationship between individual parameters and PFS and OS were performed. The correlation between tumour burden and mean tumour AD was evaluated with a scatter plot and a Spearman rank correlation with a 2-tailed test. The quantified SRETVwb from baseline PET/CT images was compared with the tumour volume quantified from PRRT cycle 1 SPECT/CT images with a scatterplot. The relative change of tumour burden from baseline to first follow-up expressed as SRETVwb, TLSREwb and measurements of the largest lesion and SUVmax was evaluated in three subgroups of ≥ 30% decrease, stable or ≥ 20% increase. PFS and OS were evaluated with Kaplan Meier survival curves comparing the subgroups and the differences between groups were tested with the log-rank test. The correlation between manual and AI-model measurements was evaluated with a scatter plot and a Spearman rank correlation with a 2-tailed test and the level of agreement was analysed with a Bland–Altman plot. A *p*-value < 0.05 was considered statistically significant. The correlation was considered very strong for Spearman’s coefficient r > 0.8, moderately strong if the value was between 0.6 up to 0.8, fair for values between 0.3 up to 0.6 and poor for values below 0.3 (Chan 2003). A sensitivity analysis with exclusion of patients who did not receive all 4 planned treatments was also performed for the primary objective.

## Results

### Patient characteristics

In total, 31 patients were included in the study where of 15 females. Median age at start of treatment was 70 years and the median time since diagnosis, was 2.5 years. The median follow-up time was 21.4 months. Seventeen patients had received previous treatment with chemotherapy. Twelve of the patients showed no impact on their performance status (ECOG 0) while 17 patients were limited in their ability to engage in strenuous activity but were still able to carry out light work (ECOG 1). Of the 19 patients with Ki-67 index within the range of 3–20%, 10 patients had a Ki-67 index of ≥ 10%. During the study 22 patients had radiological or clinical/biochemical progression of disease, with a median time to progression of 17.2 months, compared to the 9 patients without progressive disease who had a median follow-up of 28.7 months. During the timeframe of the retrospective study, 9 patients died, all of whom had progressive disease. Of the nine patients that died, 8 patients had radiological progression according to RECIST 1.1 before death. Of these, 3 patients developed new lesions, 2 patients had progression of target lesions, 2 patients developed both new lesions and had progression of target lesions and 1 patient had progression of non-target lesions. One patient exhibited only clinically documented progressive disease before death, as radiological assessments were conducted at a hospital not integrated with our digital radiology system and evaluation with RECIST1.1 could not be performed retrospectively for this examination. SPECT/CT data were retrospectively available for 29 patients for the first cycle of treatment. Twenty-seven patients had their SPECT/CT acquisition on day 4, one patient on day 5 and one patient on day 2. The characteristics are summarized in Table 1.

**Table 1:** Patient, tumour, treatment and follow-up characteristics

Variable	Data	Missing
Female	15	
Age when start of treatment, median (IQR)	70 (62–74)	
Time since diagnose at start of treatment, years, median (IQR)	2.5 (1.8–5.7)	
Follow up time until progression or end of study, months, median (IQR)	21.4 (12.3–28.7)	
Ki-67 category		1
< 3%	8	
3–20%	19	
> 20%	3	
Tumour grade		1
G1	9	
G2	18	
G3	3	
Primary tumour		
Small intestine	14	
Pancreas	12	
Rectum	3	
Colon	1	
Unknown (GEP-NET)	1	
Metastatic disease	30	
No metastases, extensive local disease	1	
Ongoing treatment with long-acting somatostatin analogue	24	
Earlier treatment		
Chemotherapy	17	
Other (Peginterferon alfa-2b, Everolimus, Sunitinib)	4	
Surgery	14	
Long-acting somatostatin analogue	2	
Liver embolization	4	
External radiotherapy	3	
Comorbidity		
Carcinoid heart disease	1	
Charlson comorbidity index (updated) mean (SD)	6 (0.9)	
GFR ml/min, median (IQR)	82 (74–88)	
WHO/ECOG performance status before treatment		2
ECOG 0	12	
ECOG 1	17	
Received treatments with PRRT		
Two	2	
Three	2	
Four	27	
Mean tumour AD, first cycle, Gy, median (IQR)	25 (17–48)	2
n follow-up examinations, median (IQR)	3 (1–4)	
Progression during follow-up	22	
Radiological progression (RECIST 1.1)	19	
Clinical/biochemical progression	3	
Endpoint not reached	9	
Overall survival	22	
Baseline PET		
Diagnostic CT	24	
Low-dose CT	7	
^68^ Ga-DOTA-TOC	23	
^68^ Ga-DOTA-TATE	8	
Days between baseline PET and first PRRT, median (IQR)	73 (38–87)	
Baseline CT		
Days between baseline CT and first PRRT, median (IQR)	65 (35–84)	
Follow-up PET	25	6
Diagnostic CT	25	
Low-dose CT	0	
^68^ Ga-DOTA-TOC	25	
Days between follow-up PET and last PRRT median (IQR)	53 (31–78)	
Performed after all received PRRT	23	
Performed during treatment	2	

Data are number of patients if not else specified (n = 31 total patients). Median values are presented with interquartile range (IQR) and mean values with standard deviation (SD)

### Quantification of tumour burden

With manual measurements of tumour burden, the median SRETVwb was 132 ml (IQR 61–302) at baseline and 71 ml (IQR 36–278) at follow-up PET/CT. The largest lesion diameter was 77 mm (IQR 44–101) at baseline and 73 mm (IQR 35–114) at first follow-up examination. Table 2 summarizes all quantitative data from baseline and follow-up PET/CT. The patient characteristics and quantitative data from baseline and follow-up PET/CT are also compared for patients with stable disease (n = 9) with the patients with progression of disease (n = 22) in supplementary table A.

**Table 2:** Quantitative values of tumour burden at baseline and follow-up PET/CT, values are median with interquartile range (IQR). Total tumour burden measured as whole-body somatostatin receptor expressing tumour volume (SRETVwb) and total lesion somatostatin receptor expression (TLSREwb)

Quantification of tumour burden	Manual measurement	Missing
Baseline PET/CT		
SRETVwb, ml (IQR)	132 (61–302)	
TLSREwb (IQR)	3684 (1522–5669)	
Largest lesion diameter, mm (IQR)	77 (44–101)	
SUVmax (IQR)	50 (26–82)	
Follow-up PET/CT		6
SRETVwb, ml (IQR)	71 (36–278)	
TLSREwb (IQR)	2251 (647–3796)	
Largest lesion diameter, mm (IQR)	73 (35–114)	
SUVmax (IQR)	41 (22–61)	
Relative change SRETVwb % (IQR)	− 26 (− 49 to 4)	
Relative change TLSREwb % (IQR)	− 35 (− 72 to − 14)	
Relative change largest lesion diameter % (IQR)	− 9 (− 20 to 3)	
Relative change highest SUVmax % (IQR)	− 26 (− 39 to − 12)	

### Prediction of PFS and OS after treatment with [177Lu]Lu-DOTA-TATE

**Fig. 2:**
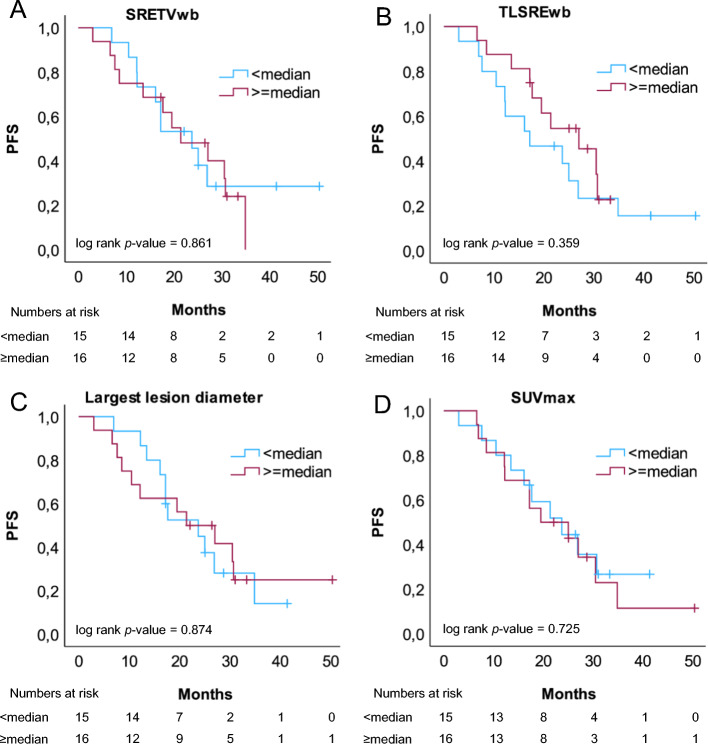
Kaplan Meier curves of PFS for high or low tumour burden at baseline PET/CT. Patients were dichotomised in two groups depending on median value of tumour burden evaluated as **A**; whole-body somatostatin receptor expressing tumour volume (SRETVwb, ml, median = 132 ml) **B**; whole-body total lesion somatostatin receptor expression (TLSREwb, sum of all lesions SUVmean*ml, median = 3684), **C**; largest lesion diameter (median = 77 mm) and **D**; SUVmax (median = 50)

**Fig. 3:**
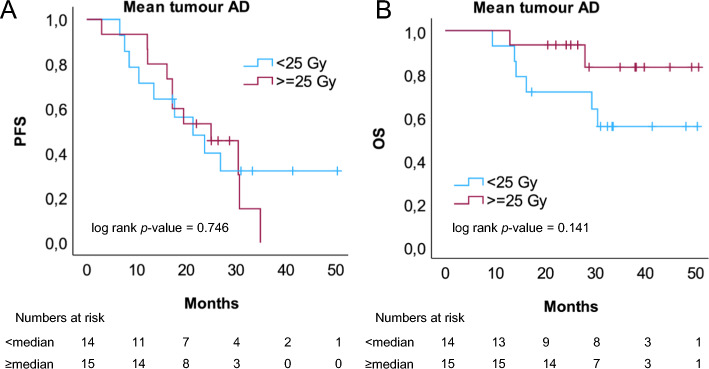
Kaplan Meier curves of PFS (**A**) and OS (**B**) for patients receiving < 25 Gy or ≥ 25 Gy. Mean tumour AD was dichotomised by the median (25 Gy) at first cycle of PRRT

Measurements of SRETVwb, TLSREwb, largest lesion diameter and the highest SUVmax quantified from baseline SSTR PET/CT, dichotomized by the median, showed no predictive value for PFS when evaluated with Kaplan Meier curves and log rank test (Fig. 2). Nor was there statistical evidence of predictive value of SRETVwb, TLSREwb, largest lesion diameter or the highest SUVmax in relation to OS (Supplementary Fig. A). Continuous variables of SRETVwb, TLSREwb, largest lesion diameter, SUVmax, mean tumour AD (Gy) and background parameters were assessed with a univariate Cox regression analysis to determine the effect of each factor on PFS and OS. No significant correlations were observed, although a tendency for worse PFS or OS was observed for Ki-67 > 3%, ECOG performance status of ≥ 1 or prior chemotherapy (Table 3). A sensitivity analyses for the primary objective with exclusion of the 4 patients who did not receive all four treatments (due to side effects or progression of disease) revealed no significant differences compared to the analyses of all patients (data not shown). When PFS and OS were evaluated with Kaplan Meier and log rank test for mean tumour AD, dichotomized by the median of mean tumour AD at first cycle of treatment, no significant differences between the groups receiving a lower or higher tumour AD was found, but there was a tendency to better survival for patients receiving a mean tumour AD of more than 25 Gy (median), as illustrated with the Kaplan Meier curves (Fig. 3B).

**Table 3:** Univariate cox regression analysis for PFS and OS

Variable	Number of patients	PFS Univariate Cox Regression Analysis			OS Univariate Cox Regression Analysis		
		HR	95% CI	*p*	HR	95% CI	*p*
SRETVwb, continuous	31	0.999	0.998–1.001	0.399	1.000	0.997–1.002	0.835
TLSREwb, continuous	31	1.000	1.000–1.000	0.461	1.000	1.000–1.000	0.754
Largest tumour lesion, diameter	31	0.999	0.987–1.011	0.859	1.001	0.983–1.019	0.924
Highest SUVmax	31	1.005	0.993–1.018	0.418	1.005	0.988–1.023	0.572
Mean tumour AD, Gy, continuous	29	0.956	0.669–1.367	0.805	0.988	0.950–1.027	0.539
Male	16	Ref			Ref		
Female	15	1.233	0.523–2.908	0.632	1.081	0.289–4.052	0.908
Age (y) < 70	15	Ref			Ref		
Age (y) ≥ 70	16	0.775	0.333–1.804	0.554	1.485	0.397–5.545	0.557
Ki-67 < 3%	8	Ref			Ref		
Ki-67 > 3%	22	2.655	0.881–7.997	0.083	3.755	0.468–30.117	0.213
ECOG = 0	12	Ref			Ref		
ECOG = 1	17	1.727	0.686–4.348	0.246	6.851	0.856–54.837	0.070
No earlier chemotherapy	14	Ref			Ref		
Earlier chemotherapy	17	1.989	0.817–4.840	0.130	3.947	0.810–19.227	0.089
Years since diagnosis < 2.5	15	Ref			Ref		
Years since diagnosis ≥ 2.5	16	1.203	0.510–2.836	0.673	0.379	0.094–1.523	0.172

### Correlation between tumour burden and mean tumour AD

**Fig. 4:**
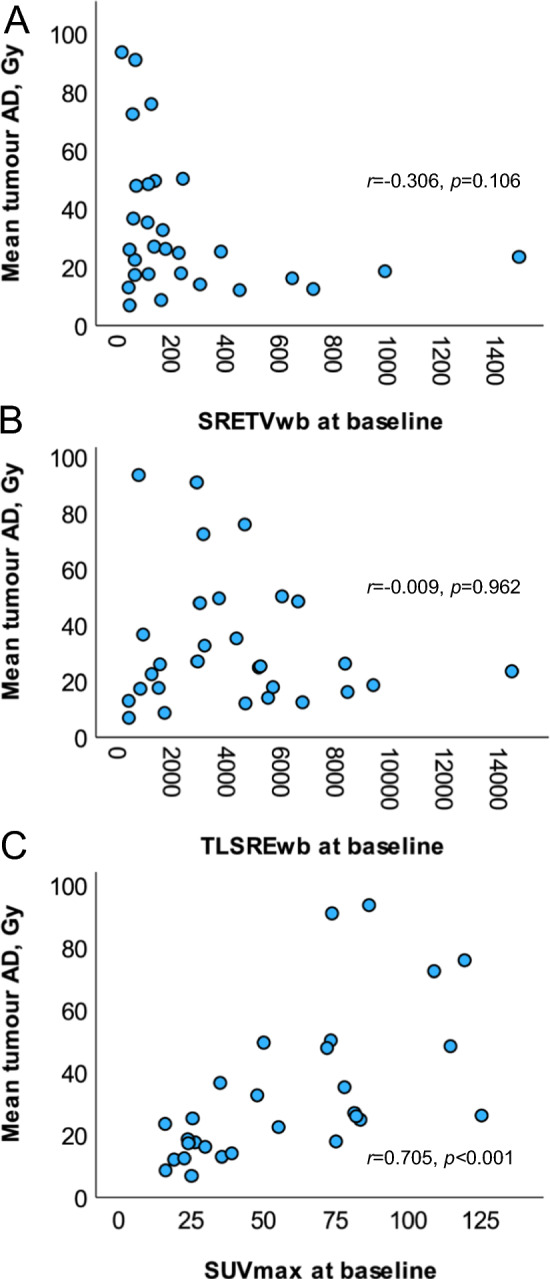
Mean tumour absorbed dose (AD), Gy, evaluated with scatterplots and Spearman rank test in correlation to baseline PET/CT parameters; **A** whole-body somatostatin receptor expressing tumour volume (SRETVwb, ml), **B** whole-body total lesion somatostatin receptor expression (TLSREwb, sum of all lesions SUVmean*ml), **C** highest SUVmax in tumour

The mean value of mean tumour AD at first cycle of PRRT was 33 Gy, median 25 Gy with a range of 7–94 Gy. Mean tumour AD showed a tendency to a weak negative correlation with SRETVwb at baseline PET/CT when evaluated with Spearman rank test, although not significant (*r* = − 0.306, *p* = 0.106). The scatterplot shows that no patients with tumour burden > 400 ml received a mean tumour AD > 30 Gy. We found no correlation between TLSREwb and mean tumour AD (*r* = − 0.009, *p* = 0.962) but instead there was a moderately strong correlation between highest SUVmax in tumour and mean tumour AD (*r* = 0.705, *p* < 0.001), illustrated in Fig. 4. The relationship between the tumour volume quantified from baseline PET/CT and from SPECT/CT images after PRRT cycle 1 is shown in Supplementary Figure B. One patient was an extreme outlier showing larger tumour burden at SPECT/CT than at PET/CT which was explained by progression of disease between baseline PET and treatment initiation.

### Impact of the relative change of tumour burden at follow-up

**Fig. 5:**
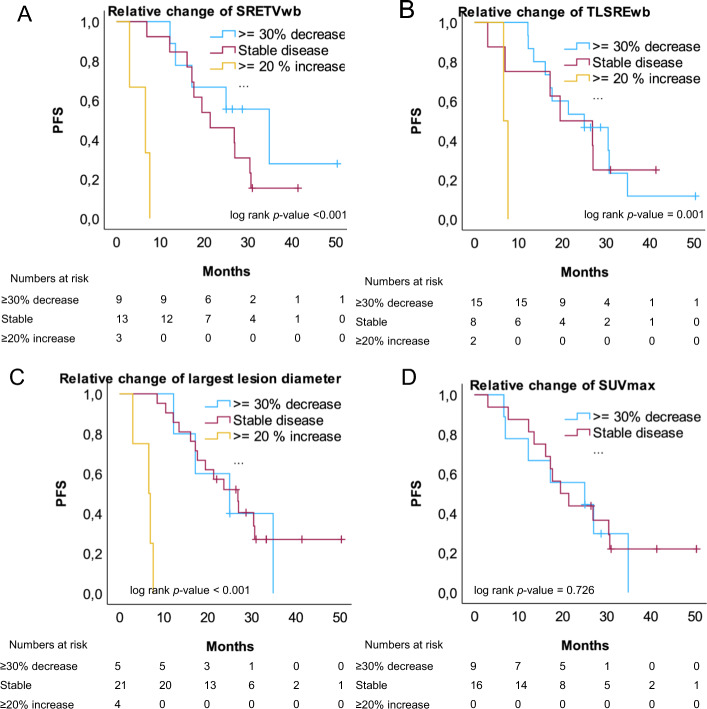
Kaplan Meier curves of PFS, evaluating the relative change of whole-body somatostatin receptor expressing tumour volume (SRETVwb, ml), whole-body total lesion somatostatin receptor expression (TLSREwb, sum of all lesions SUVmean*ml), largest lesion diameter and highest SUVmax. Patients were grouped according to the relative change evaluated in three categories ≥ 30% decrease, stable or ≥ 20% increase

PFS was significantly shorter for patients with a relative increase of tumour burden at follow-up, evaluated as SRETVwb (*p* < 0.001), TLSREwb (*p* = 0.001) and largest lesion diameter (*p* < 0.001), compared to patients with stable disease or decreasing tumour burden (Fig. 5). The results were similar for OS regarding TLSREwb (*p* < 0.001) and largest lesion (*p* < 0.024) and with a tendency but not significant for SRETVwb (*p* < 0.098) (Supplementary Fig. C). Unsurprisingly, the Kaplan Meier curves revealed that the sub-group of patients with an increase of SRETVwb, TLSREwb and largest lesion had the worst outcome after treatment. The sub-groups with a relative decrease of ≥ 30% of SRETVwb, TLSREwb and the largest lesion diameter or the group with ‘stable disease’ at first follow-up (< 20% increase or < 30% decrease) had a similar outcome. The relative change of highest SUVmax evaluated in two groups as ≥ 30% decrease or ‘stable disease’ with < 20% increase or < 30% decrease did not discriminate any significant differences in PFS (Fig. 5) or OS (Supplementary Fig. C) when evaluated with Kaplan–Meier and log-rank test. No patients had a relative increase of SUVmax of > 20%.

### Correlation between manual measurements and AI model measurements

For 7 patients the AI model missed larger lesions in the liver at baseline illustrated in Supplementary Fig. D. The Spearman rank correlation between manual and AI model measurements of SRETVwb was moderately strong with r = 0.600, *p* < 0.001 and fair for TLSREwb r = 0.525, *p* = 0.002. The median SRETVwb calculated by the AI model at baseline was 75 ml (IQR 51–193) compared to 132 ml (IQR 61–302) for manual measurements. The mean difference between manual and AI model measurements was 132 ml and with a median value of 10 ml (IQR − 4 to 40). Except for the 7 patients described above, the AI segmentation was well correlated with manual measurements. The level of agreement is illustrated with Bland–Altman plots in Supplementary Fig. E. Supplementary Table 2 are summarizing the quantitative data for the AI model from baseline and follow-up PET/CT.

## Discussion

In this small retrospective study, we could not find any predictive value of baseline tumour burden for prediction of PFS or OS after PRRT with [^177^Lu]Lu-DOTA-TATE, neither assessed as SRETVwb, TLSREwb, SUVmax nor the largest tumour lesion diameter. The published data regarding both the prognostic value of tumour burden and the predictive value of tumour burden in relationship to PRRT are scattered. Some studies have shown that a large tumour burden measured at SSTR PET/CT has prognostic value and correlates with disease progression and increased risk of disease specific mortality (Toriihara et al. 2019; Tirosh et al. 2018; Thuillier et al. 2022). A review and meta-analysis evaluated eight studies investigating the prognostic value of SRETVwb (Hou et al. 2021). Only two studies evaluated total somatostatin receptor expressing tumour volume as a predictive factor in relationship to only PRRT (Pauwels et al. 2020; Ortega et al. 2021). Pauwels et al. performed a post-hoc analyse of a previous prospective study including 43 patients who received PRRT with [^90^Y]Y-DOTATOC, and found that a baseline tumour volume of > 578 ml was associated with poorer OS on univariate analysis but not on multivariate analysis (Pauwels et al. 2020). Ortega et al. prospectively evaluated different quantitative parameters in 91 patients from [^68^ Ga]Ga-DOTA-TATE PET/CT images before and during PRRT and showed that a higher grade of SSTR expression measured as mean SUVmax in target lesions was correlated to longer PFS, but baseline volumetric parameters was not correlated to outcome, similar to our results (Ortega et al. 2021). Although, it is of interest to note that those studies are using different methods to measure tumour volume, which might affect the results, in fact the two methods used by Ortega et al. resulted in a tumour volume almost twice as high if liver was used as threshold compared to when the spleen was used as threshold, indicating large uncertainty in their segmentations (Ortega et al. 2021). In the treatment arm with high-dose octreotide in the NETTER-1 study, patients with high tumour load in the liver had shorter PFS. Although no difference was observed neither for PFS nor OS in the [^177^Lu]Lu-DOTA-TATE treatment arm depending on the tumour load in the liver, instead the lack of a large tumour lesion anywhere in the body (> 30 mm in diameter) was associated with longer PFS for the group receiving PRRT (Strosberg et al. 2020). In contrast to the NETTER-1 study a retrospective study found shorter OS if tumour load in the liver was > 25% (Ezziddin et al. 2014). A recently published study by Lee et al. retrospectively evaluated 94 patients with NET receiving [^177^Lu]Lu-DOTA-TATE therapy, showing worse PFS and OS for patients with tumour volume > 325 ml (Lee et al. 2024). Our data did not allow for using this cut-off as the number of patients with high SRETVwb was limited. The estimate for the baseline total receptor expression as TLSREwb did not show significant prognostic nor predictive value evaluated by Ebbers et al. (2021) and Toriihara et al. (2019) but showed predictive value in one study by Werner et al. (2016). Possibly the heterogeneity of tumours affects those conflicting results. Also, selection bias of patients accepted for PRRT might be a possible explanation. Outcome might differ depending on the primary location (Kipnis et al. 2021) as well as tumour grade (Sorbye et al. 2020) and a patient with a small tumour burden but rapidly progressive disease and a patient with a larger tumour burden but more indolent tumour growth might not respond equally, independent of tumour burden. In this small study we could not adjust for tumour grade or primary location but this would be of interest in a larger study. We did not find any correlation between the highest SUVmax and PFS/OS which is consistent with several other studies not finding any significant correlation between SUVmax and PFS/OS (Werner et al. 2016; Gabriel et al. 2019; Soydal et al. 2016) although another few studies also have found a correlation between SUVmax and response to treatment (Ortega et al. 2021; Kratochwil et al. 2015; Haug et al. 2010; Sharma et al. 2019). A potential explanation for the discrepant results might be tumour heterogeneity and selection bias due to patient selection for PRRT, where the pre-treatment uptake in tumour is already evaluated for eligibility of [^177^Lu]Lu-DOTA-TATE. An obstacle with SUVmax is also that no clear cut-offs have been established with different cut-offs being proposed (Kratochwil et al. 2015; Sharma et al. 2019). In the univariate Cox regression analysis Ki-67 > 3%, ECOG performance of ≥ 1 and earlier treatment with chemotherapy showed tendency to worse PFS or OS although not significant, which is in keeping with earlier studies (Albertelli et al. 2021). Ki-67 index has earlier showed both prognostic (Pape et al. 2008) and predictive value (Ezziddin et al. 2014; Aalbersberg et al. 2019) in relation to response to PRRT. Some possible reasons why Ki-67 index was not statistically significant in this study might be a change in proliferative activity since diagnosis (median 2.5 years) and evaluation of tumours with new biopsies are not routine before PRRT at our hospital, as well as the small sized study. Tumour heterogeneity is frequent in NET and there is often a variability in tumour biology between primary tumours and metastases (Reccia et al. 2023), which is one reason why dual imaging with both FDG and SSTR PET/CT is appealing (Chan et al. 2017).

The levels of AD to all tumours required to induce beneficial treatment effect during PRRT remain to be established. The median of mean tumour AD in cycle 1 was in our study found to be 25 Gy compared to the median tumour dose of 33 Gy in a recently published study by Mileva et al. (2024). With a cut-off of minimal absorbed dose of 35 Gy they found a longer PFS for patients receiving higher doses. We did not find any significant difference regarding PFS comparing the groups receiving doses of more or less than the median, but we found a tendency for better OS for patients receiving higher doses evaluated with Kaplan Meier curves, although not significant. It would be of interest to evaluate if patients receiving higher mean tumour AD have longer PFS or OS in larger prospective studies. In this small retrospective study, none of the patients with a tumour volume of > 400 ml received a higher mean tumour AD than > 30 Gy. No conclusions can be drawn from this, but further explorations are warranted. Could patients with a large tumour volume benefit from higher injected activity, maybe in the first cycles of treatment? Ezziddin et al. (2012) found a positive correlation between SUVmax > 25 and high tumour AD which is in line with our results with a significant and moderately strong correlation between SUVmax and mean tumour AD. This emphasizes the importance of high uptake at baseline SSRT PET/CT for selection of patients for treatment with PRRT. A significant variability of the uptake of [^177^Lu]Lu-DOTA-TATE has been observed both in NET tumour lesion uptake and in normal organs (Cremonesi et al. 2006). A research group in Uppsala evaluated the AD to pancreatic NETs and investigated the tumour-absorbed dose–response relationship in patients treated with PRRT (Ilan et al. 2015). They found a correlation between tumour reduction in pancreatic NETs and AD to tumour (Ilan et al. 2015) although these results could not be replicated for small intestine tumours (Jahn et al. 2020). A low mean tumour AD calculated from SPECT/CT images might serve as a negative predictive factor and those patients might benefit from earlier follow-up or different therapy targeting tumours not responding to PRRT.

As expected, we found that patients with an increasing tumour volume (SRETVwb) at follow-up had shorter PFS and the same tendency was also seen for OS although not significantly, which might be explained by too few cases. These results correlate to a newly published study showing a significantly better PFS for patients with a decrease of 10% or more in the tumour volume measured in a maximum of 5 lesions per patient (Mileva et al. 2024). Also, we found that a relative increase in TLSREwb discriminated patients with shorter PFS and OS. However, since TLSREwb is the sum of the products of SUVmean*volume for all tumour lesions interpretation of this value may be difficult since an increase could be caused by either of the variables. No patient had a ≥ 20% relative increase of SUVmax at the follow-up PET/CT. When evaluating the relative change of SUVmax we could not show any significant differences in outcome regardless of whether SUV decreased ≥ 30% or remained stable, which is consistent with previous findings of changes of SUVmax (Haug et al. 2010; Gabriel et al. 2009).

Lastly, we could not show that an in-house developed AI model could provide adequate measurements of the total tumour burden. The AI-measured tumour burden was less well correlated with manual measurements in this study than in a previous study (Gålne et al. 2023), which highlights the need for large training sets and accurate validation of new AI tools before use in clinical practice. A potential explanation why the AI model showed poorer correlation to manual measurements compared to the previous study might be that large and heterogeneous liver tumours might have been too few in the training data compared to the prevalence in this cohort.

A clear weakness of our study is its limited size and retrospective nature. The small sample size might lead to an underpowered study, and it would be of value to analyse baseline tumour burden in a larger cohort with a PET/CT acquired in closer relation to the treatment. One advantage is that we tried to include all GEP-NET patients who received [^177^Lu]Lu-DOTA-TATE at our hospital within the defined time frame. Unfortunately, there were many patients who had a PET/CT older than 6 months, of reasons possibly related to the COVID-19 pandemic, why these patients were excluded from the analysis according to predefined exclusion criteria. PET/CT performed less than 6 months before PRRT might also be inaccurate regarding measurements of the tumour burden and in one case there was obvious tumour progression from the baseline PET/CT to the SPECT/CT performed after the first cycle of PRRT. However, this patient was also concluded with progression of disease after 2 cycles of treatment and was excluded in the sensitivity analyses, which essentially gave the same results as the main analysis. In an optimal reality the PET/CT should have been performed in close relationship to the treatment which was not the case for all the patients. We chose to exclude tumours of other origin than GEP-NET due to often worse prognostics with tumours of other origin (Albertelli et al. 2021). GEP-NET is also a quite heterogeneous group of tumours, but as the disease is quite rare and not all patients receive PRRT any smaller subgroups for the study would not have been feasible. Patients in this cohort did not routinely have a FDG PET/CT performed, even if they had G2 disease with high Ki-67 index, why tumour heterogeneity can’t be assessed for in this group of patients. A potential weakness is also the segmentation method used for quantifying tumour burden at baseline PET/CT. We used a semi-automatic method segmenting 50% of SUVmax and manually adjusting for separate lesions or if background uptake (mostly in the liver) is high as earlier described (Ohlsson et al. 2022). Other segmentation methods exist, such as threshold-based formula calculated from normal liver uptake (Carlsen et al. 2022), different SUVmax thresholds, edge-based segmentation algorithms (Liberini et al. 2021) or completely manual segmentation. The best method for segmentation of tumour burden has not yet been established and the possibility for finding other results than ours, if another tumour volume segmentation method had been used, cannot be excluded. The inclusion of examinations with both [^68^ Ga]Ga-DOTA-TATE and [^68^ Ga]Ga-DOTA-TOC might impact tumour segmentations marginally as the affinity for SSTR is slightly different (Reubi et al. 2000), although we do not believe that the use of only one of the tracers would have changed the results significantly as the differences when comparing the two tracers are small (Velikyan et al. 2014; Poeppel et al. 2011). A strength of this study is that we also evaluated the treatment outcomes for sub-groups receiving low or high mean tumour AD, although SPECT/CT images were missing for two patients. As SPECT/CT images only were acquired at one time point, the tumour effective half-lives could not be estimated individually but were assessed from previously published data on a similar patient cohort (Roth et al. 2021). Inevitably, this introduced some uncertainty in the estimated tumour AD, but was not critical due to the long effective half-lives of the uptake in tumours combined with comparably late imaging time-points. In our patient cohort, the primary reason for performing [^177^Lu]Lu-DOTA-TATE SPECT/CT imaging was to enable kidney dosimetry, but as two field of views were acquired this also allowed for evaluation of the majority of the tumours. Metastases in the upper parts of the torso or pelvic/femora might have been missed when calculating the tumour volume from SPECT/CT images and mean tumour AD.

## Conclusion

In this small retrospective study, we found no value of SRETVwb, TLSREwb, largest lesion diameter or highest SUVmax evaluated at baseline SSTR PET/CT for prediction of response to PRRT. There was a moderately strong correlation between highest SUVmax and mean tumour AD which is consistent with the value of a high baseline uptake at SSTR PET/CT for selection of patients to PRRT. The relative change of tumour burden at first follow-up, both evaluated as SRETVwb, TLSREwb and largest lesion diameter showed significant predictive value, and follow-up PET/CT might be of importance for evaluation of outcome after treatment with [^177^Lu]Lu-DOTA-TATE.

### Supplementary Information

Supplementary file

## Data Availability

The datasets generated and analysed during the current study are not publicly available, but available from the corresponding author on reasonable request and relevant ethical approval.
