# Supplementary material for: Retrospective evaluation of the predictive value of tumour burden at baseline [68 Ga]Ga-DOTA-TOC or -TATE PET/CT and tumour dosimetry in GEP-NET patients treated with PRRT
Source: EJNMMI Rep. 2024 Aug 8;8(1):24. doi: 10.1186/s41824-024-00210-y (PMC11306659; doi:10.1186/s41824-024-00210-y)
Supplement: Supplementary file 1 — Supplementary file [file 41824_2024_210_MOESM1_ESM.docx]

| SUPPLEMENTARY TABLE A  Characteristics for patients grouped by disease progression | | | | |
| --- | --- | --- | --- | --- |
| Variable | **Stable disease n =9 median (IQR)** | **Missing** | **Disease progression n=22 median (IQR)** | **Missing** |
| Female | 3 |  | 12 |  |
| Age | 72 (65-75) |  | 68 (62-73) |  |
| Primary tumour |  |  |  |  |
| Pancreas | 4 |  | 8 |  |
| Small intestine | 5 |  | 9 |  |
| Colon |  |  | 1 |  |
| Rectum |  |  | 3 |  |
| Unknown GEP-NET |  |  | 1 |  |
| Tumour grade |  | 1 |  |  |
| G1 | 4 |  | 5 |  |
| G2 | 3 |  | 15 |  |
| G3 | 1 |  | 2 |  |
| Ki67 |  | 1 |  |  |
| <3% | 4 |  | 4 |  |
| 3-20% | 3 |  | 16 |  |
| >20 | 1 |  | 2 |  |
| Received treatments with PRRT |  |  |  |  |
| Two |  |  | 2 |  |
| Three | 1 |  | 1 |  |
| Four | 8 |  | 19 |  |
| Average tumour dose, first cycle, Gy (IQR) | 23.5 (17.9 – 62.75) |  | 25.7 (13.8 – 36.3) | 2 |
| Absolut GFR ml/min | 80 (68-92) |  | 82 (76-89) |  |
| Years since diagnosis (IQR) | 2.5 (0.9-11.1) |  | 3.7 (1.8 -5.5) |  |
| Follow up, months, until progression or end of study (IQR) | 28.7 (23.6-37.4) |  | 17.2 (10.0-25.5) |  |
| Radiological progression |  |  | 19 |  |
| Clinical/biochemical progression |  |  | 3 |  |
| Overall survival (n) | 9 |  | 13 |  |
| Death | 0 |  | 9 |  |
| Time to death, months (IQR) |  |  | 16.2 (13.3-28.6) |  |
| WHO/Performance status before treatment |  |  |  | 2 |
| ECOG 0 | 5 |  | 7 |  |
| ECOG 1 | 4 |  | 13 |  |
| Baseline PET/CT | 9 |  | 22 |  |
| Days between baseline PET and first PRRT (IQR) | 36 (23-87) |  | 79 (50-90) |  |
| Manual measurement |  |  |  |  |
| SRETVwb, ml (IQR) | 122 (62 - 644) |  | 146 (55- 274) |  |
| TLSREwb (IQR) | 4631 (2043 - 7972) |  | 3236 (1520 - 5323) |  |
| Largest lesion diameter, ⌀ mm (IQR) | 77 (47-101) |  | 78 (42-106) |  |
| Highest SUVmax (IQR) | 50 (24 – 94) |  | 61 (26 – 83) |  |
| Follow up PET/CT (n) | 6 | 3 | 19 | 3 |
| Days between most recent PRRT and follow up PET (IQR) | 55 (37-109) |  | 49 (28-71) |  |
| Manual measurements at follow up |  |  |  |  |
| SRETVwb, ml (IQR) | 65 (28 – 120) |  | 127 (39 - 287) |  |
| TLSREwb (IQR) | 1001 (725 – 2886) |  | 2494 (602 - 3861) |  |
| Largest lesion diameter, mm (IQR) | 67 (32 – 65) |  | 76 (35 - 118) |  |
| SUVmax (IQR) | 53 (38 – 64) |  | 36 (19 - 61) |  |
| Relative change SRETVwb % (IQR) | -42 (-59 - -6) |  | -24 (-36 - 5) |  |
| Relative change TLSREwb % (IQR) | -49 (-74 - -8) |  | -36 (-75 - -16) |  |
| Relative change largest lesion diameter % (IQR) | -9 (-22 - -7) |  | -8 (-20 - 6) |  |
| Relative change of highest SUVmax | -2 (-42 - 10) |  | -26 (-38 - -15) |  |
| Other tumour markers |  |  |  |  |
| 5HIAA mmol/l baseline (IQR) | 125 (109-2696) | 4 | 481 (114 - 912) | 13 |
| 5HIAA mmol/l follow up (IQR) | 267 (56 - 563) | 5 | 332 (153 - 936) | 13 |
| Relative Δ 5HIAA %, median (IQR) | -64 (-81 - -45) | 5 | 13 (-24 - 42) | 13 |
| CgA elevated | 6 |  | 17 |  |
| CgA mmol/l baseline (IQR) | 4.3 (1.8 – 163.5) |  | 15.0 (2.7 - 52.0) |  |
| CgA mmol/l follow up (IQR) | 2.6 (1.8 – 16.4) |  | 8.1 (1.6 - 57.2) |  |
| Relative Δ CgA %, median (IQR) | -48 (-85 - -21) |  | -24 (-57 -2.6) |  |

**Supplementary Table A.** Patient, tumour, treatment, and follow-up characteristics comparing patients with stable disease with patients with progression of disease. Data are number of patients if not else specified (n=31 total patients). Median values are presented with interquartile range (IQR) and mean values with standard deviation (SD).


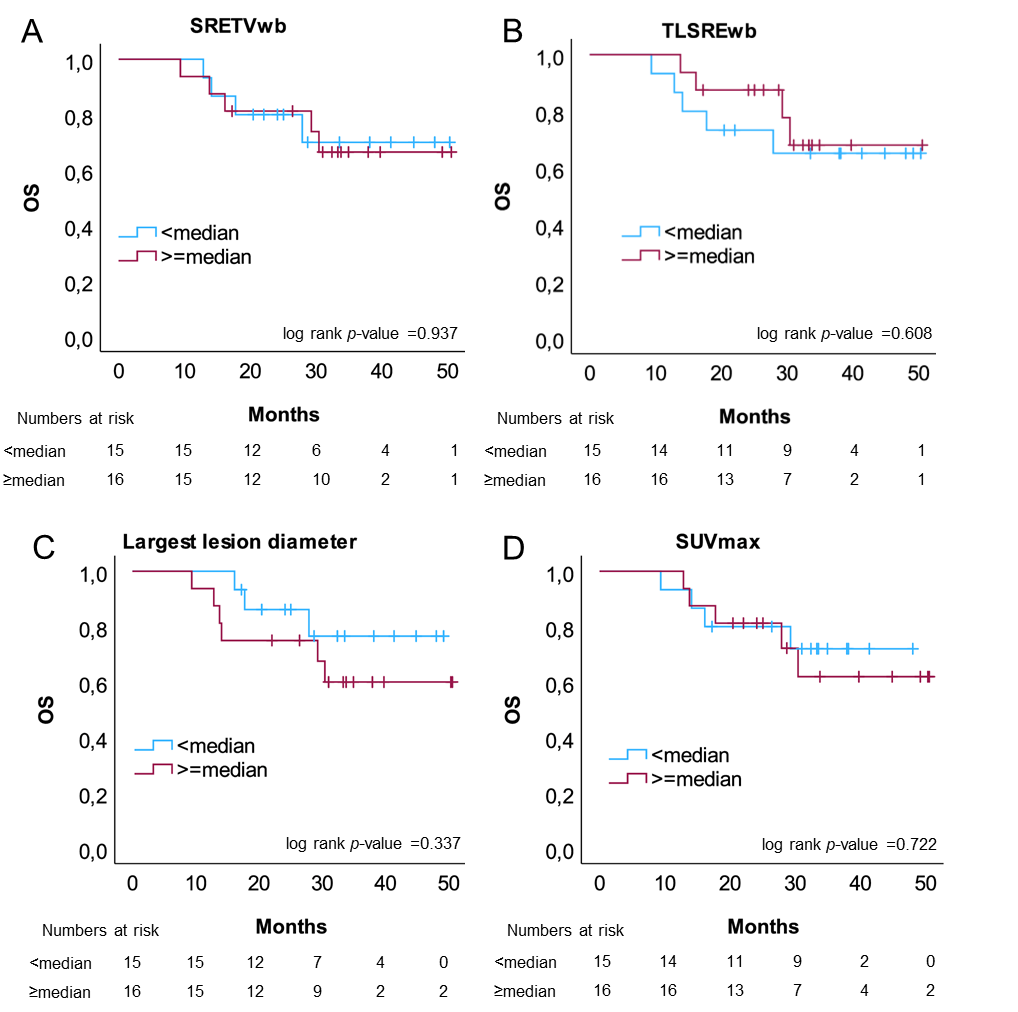


**Supplementary Figure A.** Kaplan Meier curves of OS for high or low tumour burden at baseline PET/CT. Patients were dichotomised in two groups depending on median value of tumour burden evaluated as A; whole-body somatostatin receptor expressing tumour volume (SRETVwb, ml, median = 132 ml) B; whole-body total lesion somatostatin receptor expression (TLSREwb, sum of all lesions SUVmean*ml, median = 3684), C; largest lesion diameter (median = 77 mm) and D; SUVmax (median = 50).


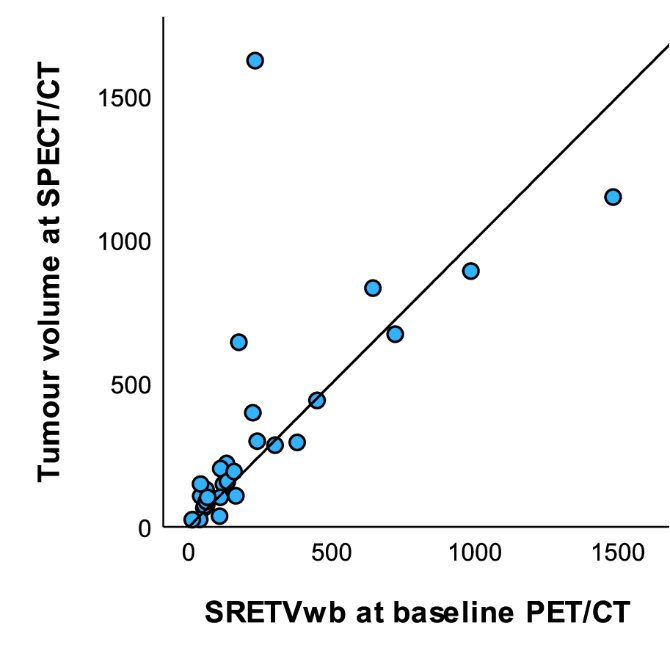


**Supplementary Figure B.** The relationship between the tumour volume quantified from baseline PET/CT and the tumour volume quantified from SPECT/CT images after PRRT cycle 1 is illustrated with a scatterplot. Line of identity. Spearman rank correlation *r*=0.825, *p*<0.001.


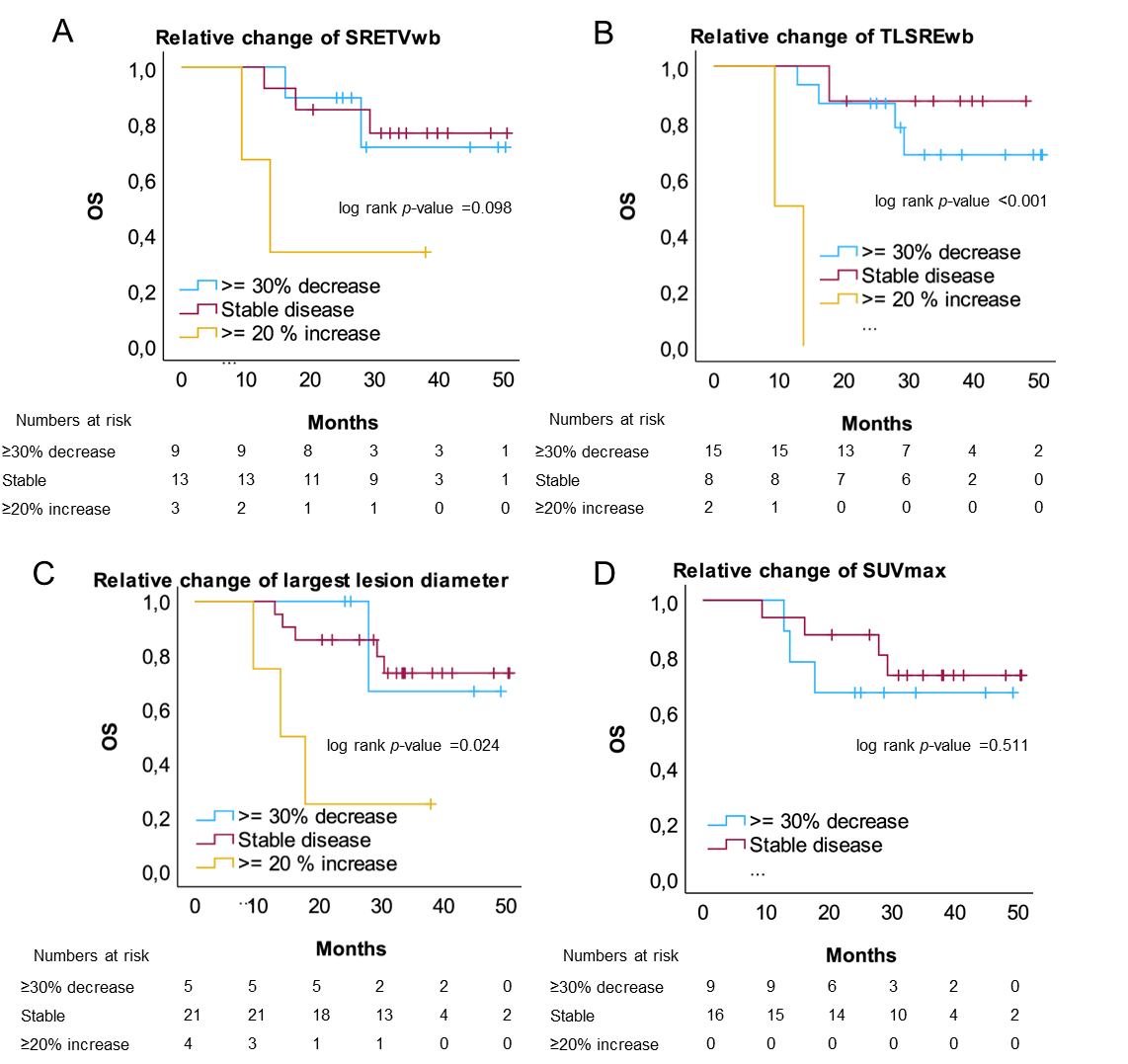


**Supplementary Figure C.** Kaplan Meier curves of OS, evaluating the relative change of whole-body somatostatin receptor expressing tumour volume (SRETVwb, ml), whole-body total lesion somatostatin receptor expression (TLSREwb, sum of all lesions SUVmean*ml), largest lesion diameter and highest SUVmax. Patients were grouped according to the relative change evaluated in three categories ≥30% decrease, stable or ≥ 20% increase.


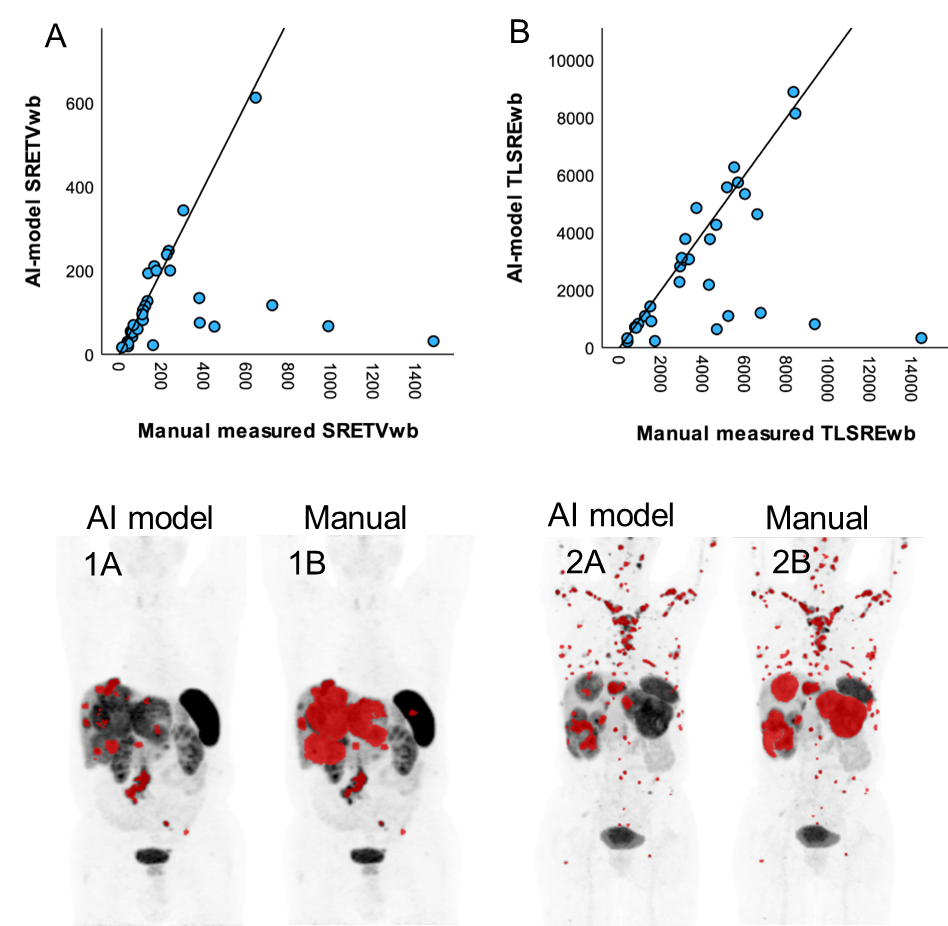


**Supplementary Figure D.** Scatterplots of AI model and manual measurements for SRETVwb and TLSREwb at baseline PET/CT. Line of identity in images. Spearman rank correlation *r*=0.6, *p*<0.001 (A) respective *r*=0.525 *p*=0.002 (B). MIP images from patient A and B with illustration of large lesions that the AI model missed, especially in the liver (1A, 2A) compared with manual segmentation (1B, 2B).


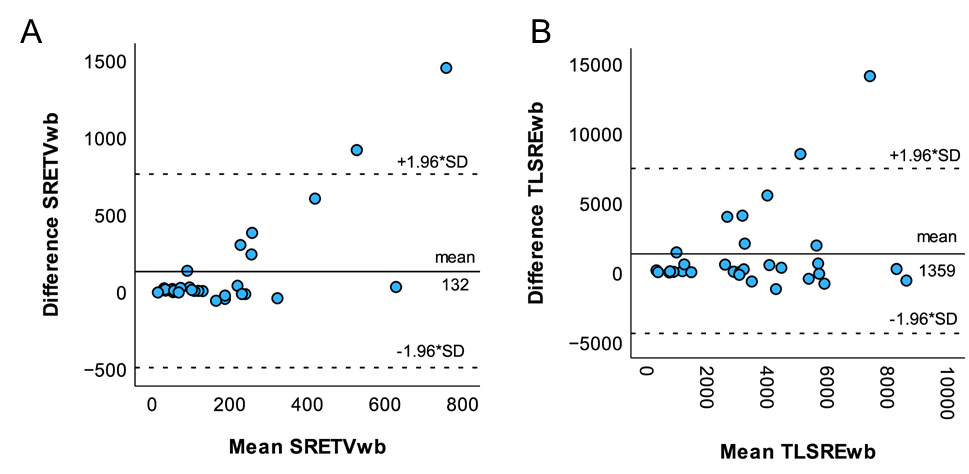


**Supplementary Figure E.** Bland Altman plots illustrating the level of agreement between manual measurements and the AI model for SRETVwb (A) respectively TLSREwb (B). Dotted lines specify the 95% upper and lower limits of agreement.

| SUPPLEMENTARY TABLE B | | |
| --- | --- | --- |
| Quantification of tumour burden | **AI-model** | **Missing** |
| Baseline PET/CT |  |  |
| n lesions IIQR) | 19 (10 - 46) |  |
| SRETVwb, ml (IQR) | 75 (51 - 193) |  |
| TLSREwb (IQR) | 2281 (816 - 4637) |  |
| Largest lesion volume, ml (IQR) | 37 (9 - 78) |  |
| SUVmax (IQR) | 50 (26-82) |  |
|  |  |  |
| Follow-up PET/CT |  | 6 |
| n lesions (IQR) | 16 (6 - 35) |  |
| SRETVwb, ml (IQR) | 47 (20 - 121) |  |
| TLSREwb (IQR) | 722 (412 - 2866) |  |
| Largest lesion volume, ml (IQR) | 16 (8 - 70) |  |
| SUVmax (IQR) | 41 (22 – 61) |  |
| Relative change SRETVwb % (IQR) | -38 (-71 - 1) |  |
| Relative change TLSREwb % (IQR) | -48 (-71 - -27) |  |
| Relative change maximal lesion volume % (IQR) | -43 (-78 - 15) |  |
| Relative change highest SUVmax % (IQR) | -26 (-39 - -9) |  |

**Supplementary Table B.** AI model derived quantitative values of tumour burden at baseline and follow-up PET/CT, values are median with interquartile range (IQR). Total tumour burden measured as whole-body somatostatin receptor expressing tumour volume (SRETVwb) and total lesion somatostatin receptor expression (TLSREwb).
